# Venous Thromboembolism in Pediatric Vascular Anomalies

**DOI:** 10.3389/fped.2017.00158

**Published:** 2017-07-24

**Authors:** Taizo A. Nakano, Chadi Zeinati

**Affiliations:** ^1^Vascular Anomalies Center, Center for Cancer and Blood Disorders, Children’s Hospital Colorado, Denver, CO, United States; ^2^Vascular Anomalies Center, Children’s Hospital Los Angeles, Keck School of Medicine of the University of Southern California, Los Angeles, CA, United States

**Keywords:** vascular malformation, venous thromboembolism, sclerotherapy, vascular tumor, vascular anomaly

## Abstract

The presence of a vascular anomaly suggests that capillaries, veins, arteries, and/or lymphatic vessels have demonstrated abnormal development and growth. Often dilated and misshaped, these vessels augment normal flow of blood and lymphatic fluids that increases the overall risk to develop intralesional thrombosis. Abnormal endothelial and lymphoendothelial cells activate hemostasis and hyperfibrinolytic pathways through poorly understood mechanisms, which contribute to the development of localized intravascular coagulopathy. Vascular malformations, tumors, and complex combined syndromes demonstrate varying degrees of prothrombotic activity and consumptive coagulopathy depending on the vessels involved and the pattern and extent of abnormal growth. The clinical impact of venous thromboembolism in pediatric vascular anomalies varies from painful syndromes that disrupt quality of life to life-threatening embolic disease. There remains little literature on the study, evaluation, and treatment of thrombosis in pediatric vascular anomalies. However, there have been great advances in our ability to image complex lesions, to surgically and interventionally augment disease, and to provide enhanced supportive care including patient education, compression therapy, and strategic use of anticoagulation.

## Introduction

Pediatric vascular anomalies affect roughly 5% of the population and consist of abnormally developed and misshaped capillaries, veins, arteries, and/or lymphatic vessels. They demonstrate irregular flow of blood and lymphatic fluids, consist of malformed and overly activated endothelial walls, and can carry an increased risk for intralesional and/or systemic thrombosis. The clinical impact of thrombosis in pediatric vascular anomalies ranges from mild episodic pain associated with small superficial thrombi to life-threatening sequela of embolic thrombi formed *via* connections to deeper ectatic vessels (1). The chronic formation and lysis of thrombi can progressively worsen pain and increase lesion size as the vessel walls lose elasticity. These complications can substantially limit daily activities and the negative impact on a child’s quality of life directly contributes to a family’s decision to pursue aggressive therapeutic interventions. A multidisciplinary approach is recommended to treat all vascular anomalies, and the morbidity and mortality associated with thrombotic complications dictates the need to include a physician familiar with pediatric hematology as part of this multidisciplinary team.

Vascular anomalies are divided between vascular malformations (congenitally malformed vessels) and vascular tumors (acquired proliferating growths of endothelial and lymphendothelial origin) (2). The International Society for the Study of Vascular Anomalies (ISSVA) has advocated for the use of this standardized nomenclature to help facilitate diagnosis and reduce treatment inconsistences (3). Although very few publications exist that study the development of venous thromboembolism (VTE) in vascular anomalies, vascular malformations, specifically venous malformations (VMs), have been shown to demonstrate a high frequency of acquired thrombosis. A number of vascular tumors have been associated with a variety of local and systemic coagulopathies. However, if present, they tend to be associated with hemorrhagic complications and to a much lesser degree thrombotic complications. The aim of the current article is to summarize the published clinical experience with and treatment of VTE in pediatric vascular anomalies, specifically highlighting those associated with VMs.

## Vascular Malformations

Vascular malformations, the abnormal congenital development of capillaries, veins, arteries, and lymphatics, have historically been difficult to diagnose and classify. Vascular malformations are now classified according to flow velocity (documented by Doppler ultrasonography) and by the predominant vessel types involved. Slow-flow (or low-flow) lesions consist of capillaries, veins, and/or lymphatics. Fast-flow (or high-flow) lesions include arteriovenous malformations and aneurysms (3, 4). Although Doppler ultrasonography plays an important role to classify a lesion, magnetic resonance imaging (MRI) is the most accurate imaging modality to define the extent of a vascular malformation and is essential to obtain prior to any interventional therapy (5). Clinical history and exam should be utilized as adjuvant tools to accurately diagnose a child’s vascular malformation.

### Venous Malformations

Venous malformations develop from congenital errors in vascular morphogenesis. Amongst all vascular malformations, the slow-flow lesions, most prominently venous, lymphovenous (LVM), and capillary-lymphovenous (CLVM) malformations, demonstrate the highest incidence of baseline coagulation abnormalities with increased risk for thrombosis in both children and adults. VMs are present at birth and can greatly vary in location, size, depth, and degree of ectasia. The defective and dilated veins demonstrate irregularly attenuated walls that, histologically, have decreased smooth muscle cells and are lined only by endothelial cells (6, 7). Skin overlying a VM often demonstrates a marked bluish discoloration often confused for a bruise at birth. They are soft and compressible on exam. VMs will demonstrate bright hyperintensity on spin-echo T2-weighted MRI sequences with fat suppression (6). The blood flow through VMs is disrupted, turbulent, and stagnant at times. Vessel injury and inflammation activate endothelial cells, activate coagulation and consumption of clotting factors, and generate thrombin and fibrin (8, 9). This unique chronic consumptive coagulopathy, coined localized intravascular coagulation (LIC), is characterized by decreased levels of plasma fibrinogen, factor V, factor VIII, factor XIII, and increased D-dimer (10). LIC increases a child’s risk for intralesional thrombosis and their peri-surgical/procedural risk for severe hemorrhage. VMs with LIC demonstrate all of the components of Virchow’s triad including stasis of blood flow, injury to vascular wall, and activation of the coagulation cascade. Virchow’s triad is widely accepted to define high risk factors that, when combined, result in the development of VTE. The extent of the VM correlates with the severity of the LIC and overall risk for thrombosis formation (6, 8).

As a child ages, VMs swell and can become bulky depending on location and a patient’s mobility. Chronic intralesional thrombosis results in further deformation of the lesion and contributes to ongoing inflammation and chronic pain. With abnormal fibrinolysis, superficial and intralesional thrombi are not adequately cleared. The chronic thrombi bind calcium deposits and form hard, round thrombi termed phleboliths. Phleboliths are often palpable, painful, easily identifiable on imaging, and can greatly impair function. Phleboliths of the lower extremities, for example, may impact a child’s ability to ambulate. On MRI, phleboliths present as low-intensity signals on T1 and T2 images.

Venous malformations can be further classified according to Puig et al. into four types based on their venographic appearance/drainage (11). Type 1 is a sequestered malformation with no discernable venous drainage to the surrounding venous system. Type 2 has small normal appearing draining veins into the superficial or deep venous system. Type 3 has enlarged and ectatic draining veins. In Type 4, the malformation itself consists of dilated venous ectasia. The presence of ectatic vessels that connect to the deep venous system conveys an increased risk of deep vein thrombosis (DVT) and the sequela of thrombotic emboli including pulmonary embolus (PE). Thrombosis in a Type 1 or 2 lesion is unlikely to cause a DVT or PE compared to the higher risk demonstrated in Type 3 and 4 lesions. MR venography, ultrasonography, and direct venography may be helpful to demonstrate these dangerous connections to the deep venous system. The degree of functional impairment, the severity of pain, and the risk of potential deep embolic VTE are used to determine the need for invasive therapeutic interventions such as sclerotherapy or vessel embolization (12).

A positive D-dimer can be found in 42% of patients with a VM (13). This is close to five times higher than those with other non-venous vascular malformations (14). As a surrogate marker for thrombin activation, the D-dimer already plays a prominent role in the diagnostic work up for suspected thromboembolism in pediatric and adult medicine. However, the D-dimer is uniquely useful to identify a venous component of complex or unclear vascular malformations. Sepulveda et al. carried out one of the few studies focused on pediatric patients with vascular malformations complicated by thrombosis. The authors suggested clinical and laboratory factors associated with higher risk of thrombotic complications include the extent of the malformation, presence of palpable phleboliths, increased age, and elevated D-dimer (15). A child’s platelet count can be mildly decreased in LIC, which helps distinguish the LIC of VMs from the severely low platelet counts demonstrated in the Kasabach–Merritt phenomenon (KMP), a coagulopathy specific to the vascular tumors kaposiform hemangioendothelioma (KHE) and tufted angioma (TA) (see Vascular Tumors) (8). The coagulopathy in VMs has been shown to worsen with interventional procedures such as sclerotherapy and embolization, consisting of decreased platelets, a drop in fibrinogen, and the conversion from a negative to positive D-dimer (14).

### Complex Combined Vascular Malformation Syndromes

Complex combined vascular malformation syndromes, like Klippel–Trenaunay syndrome (KTS), are associated with an increased frequency of VTE. It is the venous and venolymphatic components of these lesions that demonstrate the increased thrombophilia. The thrombi in these complex combined syndromes range from superficial and minimally symptomatic phleboliths to large vessel life-threatening DVTs and PEs. Blood flow is perturbed and slowed through varying degrees of ectatic vessels that activate the local coagulation system and results in the formation of recurrent thrombi (16).

Klippel–Trenaunay syndrome is a rare congenital vascular malformation syndrome characterized by a combination of capillary, lymphatic, and VMs (CLVM) associated with localized abnormal overgrowth of bone and/or soft tissue that often results in limb hypertrophy. Patients with KTS commonly demonstrate a mild coagulopathy and an elevated D-dimer that develops from extensive clumps of slow-flow malformation. These patients are prone to both bleeding and thrombotic complications. DVTs are life-threatening complications reported to occur in 8–22% of KTS patients (16–18). KTS patients can demonstrate rudimentary deep venous systems with persistent embryonal veins such as the lateral vein of servelle or lateral marginal vein in the affected lower extremity and a persistent sciatic vein (19). These veins predispose to stasis and cannot be removed since the deep system is not developed and removal will place the patient at risk of venous hypertension. Enlarged ectatic veins connected to central veins place the patient at risk of developing PE. A specific incidence of PE is difficult to be reported as the literature consists of case reports and case series (20–22). One study reports an incidence of 4% PE and 4% DVT (23). Chronic thromboembolic pulmonary hypertension results from incomplete resolution of the vascular obstruction caused by PE. It tends to occurs at an older age in patients with KTS or large VMs after a history of recurrent PE (24). To adequately monitor for chronic pulmonary complications, a pediatric pulmonologist should be involved in the multidisciplinary care of KTS patients.

The development of thrombosis occurs at greater frequency post-operatively, post-procedurally, and after trauma. A large cohort of KTS patients studied by Oduber et al. compared children with KTS to young–adult controls and demonstrated higher D-dimer levels (*p* < 0.001) in KTS patients. Additionally, the median levels of protein C (*p* = 0.003) and protein S (*p* = 0.01) were significantly lower in the KTS patients (21). The D-dimer can be used to help differentiate complex combined lesions with predominantly slow-flow VMs from lesions with predominantly fast-flow arteriovenous malformations like Parkes–Weber syndrome (25).

Proteus syndrome is characterized by an asymmetric overgrowth of body parts, vascular malformations, epidermal nevi, and dysregulated adipose tissue. It is associated with mutations in the AKT1 gene and PTEN mutations. Patients with Proteus syndrome carry an increased risk for DVT and PE, likely secondary to stagnant flow in the dilated anomalous veins in the affected limb (26).

CLOVES syndrome (congenital lipomatous overgrowth, vascular malformations, epidermal nevi, skeletal/scoliosis abnormalities) is part of the PIK3-CA-related overgrowth syndromes. Patients with CLOVES syndrome may have abnormalities in central conduction with central and thoracic phlebectasia that places them at higher risk for PE and sudden death, especially during procedures (27, 28).

## Vascular Tumors

Vascular tumors are abnormal proliferations of cells of vascular origin. They are divided between benign hemangiomas (including infantile and congenital), the more aggressive and infiltrating KHE and TA, and a rare category of malignant vascular tumors that includes angiosarcoma (29). Infants with a benign infantile hemangioma (IH) may demonstrate a slightly elevated platelet count, D-dimer, and a slightly lower fibrinogen level. However, these infants neither develop clinically symptomatic coagulopathies or VTE. Congenital hemangiomas, which are histologically distinct from IH, are divided into a rapidly involuting (RICH), non-involuting (NICH), or partially involuting congenital hemangiomas. The RICH subtype may demonstrate a mild coagulopathy consisting of thrombocytopenia, low fibrinogen, elevated D-dimers, and fibrin degradation products. This coagulopathy, however, is typically self-limited and not associated with bleeding complications (30).

Kaposiform hemangioendothelioma is a vascular neoplasm of infancy and childhood that, unlike classic IHs, does not undergo spontaneous involution. It can develop a life-threatening coagulopathy known as the KMP, which includes severe thrombocytopenia, hypofibrinogenemia, and consumptive coagulopathy (31). The cause of this coagulopathy has been theorized to be secondary to trapping and consumption of inappropriately activated platelets combined with hyperfibrinolysis (2). Unlike the thrombosis risk associated with the LIC of venous and venolymphatic malformations, vascular tumors with KMP carry a disproportionate increased risk of severe hemorrhage and is associated with a 10–30% mortality risk (32). Cause of death is often related to hemorrhage, functional impairment, high output cardiac failure, and shock.

## Therapy

There are no established evidence-based guidelines to prevent and treat VTE in children with vascular anomalies. Individual and consensus practice publications have described mostly supportive care measures as there have been no prospective pharmacotherapy trials to address VTE in pediatric vascular anomalies. Multidisciplinary approach is important to proper treatment planning and better outcomes.

Patients with slow-flow vascular malformations (VM, LVM, or CLVM) should undergo evaluation by a pediatric hematologist for baseline screening, peri-operative management, and/or pregnancy planning. Laboratory evaluation, at minimum, should include complete blood cell count, measurement of prothrombin time, activated partial thromboplastin time, plasma D-dimer, and fibrinogen. Guided by clinical suspicion, additional thrombophilia labs to consider include serum protein C and S levels, antithrombin levels, and screening for the prothrombin gene and Factor V Leiden gene mutations. Patients and families should be provided education on the signs and symptoms of thrombosis and thromboembolic complications. Practitioners caring for patients with high-risk lesions should have a low threshold for diagnostic testing. Patients with extensive slow-flow lesions and complex combined lesions require long-term monitoring for thrombotic complications (12). Patients should be advised to avoid additional prothrombotic risk factors including obesity and the use of estrogen-based oral contraceptives (17).

The use of well-fitted high-quality, elastic compression garments has become a standard of care in most vascular malformations that include a venous component (VM, LVM, CLVM). When used appropriately, the compression garment acts to reduce transmural pressure, prevent venous stasis, limb swelling, and can decrease LIC (7, 8). The result is reduced phlebolith formation and decreased pain. Data to support the benefits of compression garments are primarily extracted from adult studies that demonstrate reduced rates of post thrombotic syndrome in patients with VTE (33).

There has been a long-standing, but poorly studied, role for anticoagulation in the treatment of slow-flow vascular malformations. The strategic administration of low-molecular-weight heparin (LMWH) has been used in attempts to suppress LIC, drive down elevated D-dimers, and essentially “calm down” the over activated coagulopathy and consumption demonstrated in many VMs. Unfortunately, the field has yet to carry out adequate prospective trials to study the efficacy and safety of anticoagulation in this population. Furthermore, the field has yet to develop consensus criteria to guide when anticoagulation use is indicated, what dose should be administered, and how long therapy should continue. Factors commonly evaluated in the decision to initiate anticoagulation include the degree and type of venous ectasia and laboratory evidence of LIC including elevated D-dimer and reduced/consumed fibrinogen.

Common practice is to consider use of LMWH to prevent complications of hemorrhage or thrombosis during and after interventional or surgical procedures and to alleviate the symptoms of pain associated with VM thrombosis. To prevent peri-procedural complications, prophylactically dosed LMWH (0.5 mg/kg/dose once or twice a day subcutaneously) is often administered for at least 2 weeks prior to a procedure and is continued for an additional 2 weeks post-procedure (34). Short courses (2–3 weeks) of prophylactically dosed LMWH are often administered to alleviate the pain caused by inflammation and thrombosis/phleboliths occurring within extensive VMs. Depending on phlebolith location, they can be quite painful and greatly impact a child’s quality of life. Even when prophylactically dosed, poor drug clearance is theoretically possible in extensive malformations and it remains undetermined if and how often serum LMWH anti-Xa levels should be monitored. Patients that receive prolonged exposure to LMWH should have blood counts checked routinely and a bone densitometry (DEXA) scan should be completed to evaluate for ongoing osteopenia. Temporary IVC filter placement may be necessary in patients with high risk for DVT/PE such as CLOVES or KTS (22, 28).

There is equivocal evidence to support the use of antiplatelet agents to reduce LIC in patients with VMs. The LIC in slow-flow lesions is based on the consumption of coagulation factors and does not result in either a significantly decreased or increased platelet count. Therefore, without any prospective trials to guide safety and efficacy, providers anecdotally use aspirin and non-steroidal anti-inflammatory (NSAIDS) agents to stabilize symptoms, reduce inflammation, and decrease severity of pain episodes (35).

For children with symptomatic lesions that cause pain and/or deformity, sclerotherapy is a first-line intervention to consider. Treatment involves percutaneous, catheter-directed injection of a sclerosant such as dehydrated alcohol (ethanol) or sodium tetradecyl sulfate (Sotradecol). Injecting one of these irritants induces endothelial damage and encourages the malformed vessel to scar down on itself. Immediately after a sclerotherapy procedure, fibrinogen and platelet levels decline and D-dimer elevates higher than normal values (14). For lesions at high risk for VTE, several techniques have been described to block the draining veins, allow the sclerosant to remain in the malformation, and reduce the risk of off-target vessel exposure. Endovenous laser or radiofrequency ablation of the draining veins has been described. An additional interventional approach to block draining veins is embolization with coils, *n*-butyl-2-cyanoacrylate liquid emboli (nBCA) or onxy (36, 37). There is scant new data to support the use of cryoablation techniques for the treatment of large symptomatic VMs (38).

Surgery is also considered as a first-line therapy for the treatment of symptomatic VMs. When appropriate, surgery aims at total resection of the malformation to decrease negative impact of the lesion and decrease risk of recurrence. However, given the potential for hemorrhagic complications in extensive, ectatic VMs, surgical debulking is not always possible or only possible with adjunct sclerotherapy or nBCA/onxy embolization. Patients with underlying LIC are at risk to develop peri-operative DIC and, if the underlying lesion is not completely excised, there is an increased risk of post-operative hemorrhage. Rapid administration of blood replacement products (including fresh frozen plasma, cryoprecipitate) and antifibrinolytic agents may be indicated to stop post-operative hemorrhagic complications (39).

## Conclusion

The development of VTE in pediatric vascular anomalies greatly impacts a patient’s quality of life and influences therapeutic decisions. Early screening of high risk lesions is recommended and evaluation may include imaging, laboratory testing, and appropriate documentation of any functional impact the lesion has. With an increased awareness of the pathophysiology and clinical impact of VTE in vascular malformations, prospective trials and consensus pediatric guidelines are necessary for the field to standardize care and to determine the safety and efficacy of pharmacotherapy interventions. A multidisciplinary approach is vital to the proper management of pediatric vascular anomalies to ensure all surgical, interventional, pharmacological, and supportive care options have been considered.

## Author Contributions

TN and CZ both contributed, designed, and wrote the manuscript.

## Conflict of Interest Statement

The authors declare that the research was conducted in the absence of any commercial or financial relationships that could be construed as a potential conflict of interest.
